# Future of Three Endemic Woody Species of *Colutea* (Fabaceae) in a Changing Climate in Iran

**DOI:** 10.1002/ece3.71318

**Published:** 2025-05-09

**Authors:** Amin Zeraatkar, Elham Hatami, Farzaneh Khajoei Nasab, Najmaldin Ezaldin Hassan

**Affiliations:** ^1^ Research Division of Natural Resources Chaharmahal and Bakhtiari Agricultural and Natural Resources Research and Education Center (AREEO) Shahrekord Iran; ^2^ Department of Biology, Faculty of Science Razi University Kermanshah Iran; ^3^ Department of Plant Sciences and Biotechnology, Faculty of Life Sciences and Biotechnology Shahid Beheshti University Tehran Iran; ^4^ College of Engineering, Civil and Environment Department University of Zakho Zakho Kurdistan region Iraq

**Keywords:** conservation, global warming, legumes, MaxEnt, suitable habitat

## Abstract

Woody plants offer valuable services to ecosystems, including providing useful products, stabilizing ecosystems, and mitigating climate and pollution effects. However, they face significant abiotic and biotic stresses, with climate change being the most critical challenge. It is essential to understand that reducing populations of woody species, particularly those found only in a specific area, can have severe and irreversible effects on the entire ecosystem. Therefore, exploring the potential influence of climate change on the distribution of endemic woody species is an appealing subject for conservation researchers. This study investigates how climate change affects the distribution of three endemic species of woody plants in the genus *Colutea* in Iran. The MaxEnt model was used to analyze the data, and the results showed that the model was effective for predicting the impact of climate change on the plants (AUC ≥ 0.9). The distribution of *C. persica* was significantly affected by solar radiation, Precipitation of Wettest Month, sand, and silt content. *C. porphyrogamma*'s distribution was impacted by Mean Temperature of Coldest Quarter, Precipitation of Driest Month, and Cation Exchange Capacity, while *C. triphylla* was most affected by Precipitation Seasonality, Precipitation of Driest Quarter, and Isothermality. According to the findings, the distribution of these species is expected to decrease in the 2050s and 2070s due to climate change, based on the RCP4.5 and RCP8.5 climate scenarios. These findings can be useful for developing strategies to manage the impacts of climate change on these species.

## Graphical abstract

Investigating how climate change is influencing the distribution of three Colutea species endemic to Iran by modeling the potentially suitable distribution range of the species under current and proposed future climate change scenarios.

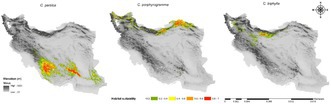

## 1 Introduction

Climate change is frequently recognized as a significant threat to biodiversity, species survival, and the stability of ecosystems across various biomes (Maclean and Wilson 2011; Bellard et al. 2012). Extreme high temperatures and prolonged drought conditions, which result in various abiotic and biotic stresses, significantly impact the physiological mechanisms of plants as well as their growth, development, reproductive success, and distribution across different geographical areas (Kumar et al. 2021). The Intergovernmental Panel on Climate Change (IPCC) declared that the number of endangered species increases notably each year and approximately 58% of plants will lose their ecological niche and natural habitats under the pressure of climate change in 2080 (Warren et al. 2013; IPCC 2014). A substantial proportion of such endangered species has been defined as endemics, typically restricted to specific geographic regions and often existing in a few small wild populations (Chichorro et al. 2019; Staude et al. 2020). Most endemic species possess a combination of characters such as limited distributions, small population sizes, unique genetic reserves, specific habitat requirements, and reduced dispersal capacities. Consequently, the presence of the abovementioned characteristics in endemic species increases their vulnerability to a high risk of endangerment or even extinction (Işik 2011; Khajoei Nasab, Mehrabian et al. 2024; Khajoei Nasab, Shakoori et al. 2024; Zeraatkar and KhajoeiNasab 2024; Liao et al. 2023).

Moreover, endemic species, particularly woody plants, may face difficulties in tracking suitable conditions or adapting quickly enough to keep up with the unprecedented rate of environmental changes (Paulsen et al. 2000; Khajoei Nasab, Mehrabian et al. 2024; Khajoei Nasab, Shakoori et al. 2024). It is estimated that around 85% of the world's forest cover has undergone different levels of degradation; in particular, the loss of forest wildlands is occurring at an alarming pace (Sefidi et al. 2022). Forests and woodlands are predominantly threatened by natural system modification, mining activities, dam construction, fire incidents, disease epidemics, diminished rainfall, elevated temperatures, drought, agricultural endeavors, and overgrazing (Akhani 2015). Furthermore, the woody endemics as multifunctional taxa (e.g., keystone, umbrella, surrogate) show more important ecological roles in ecosystems, thus making them important cases for evaluating the conservation and distribution patterns (Andelman and Fagan 2000). Southwest Asia, in particular, Flora of Iran is significantly comprised of woody plant taxa, such as trees and shrubs, which often spread on the intersection of diverse phytogeographical units (Takhtajan 1986; Zahran 2010). The Alborz and Zagros mountains are the main centers of endemism of trees and shrubs in Iran (Hedge and Wendelbo 1978; Mehrabian et al. 2020; Noroozi et al. 2016). Over the last decade, numerous studies have been conducted to determine the potential distribution of woody species and their responses to climate change in Iran (Ahmadi et al. 2020; Alavi et al. 2019; Alipour et al. 2023; Taleshi et al. 2019; Khajoei Nasab, Mehrabian et al. 2024; Khajoei Nasab, Shakoori et al. 2024; Zeraatkar and Khajoei Nasab 2024).

**FIGURE 1:**
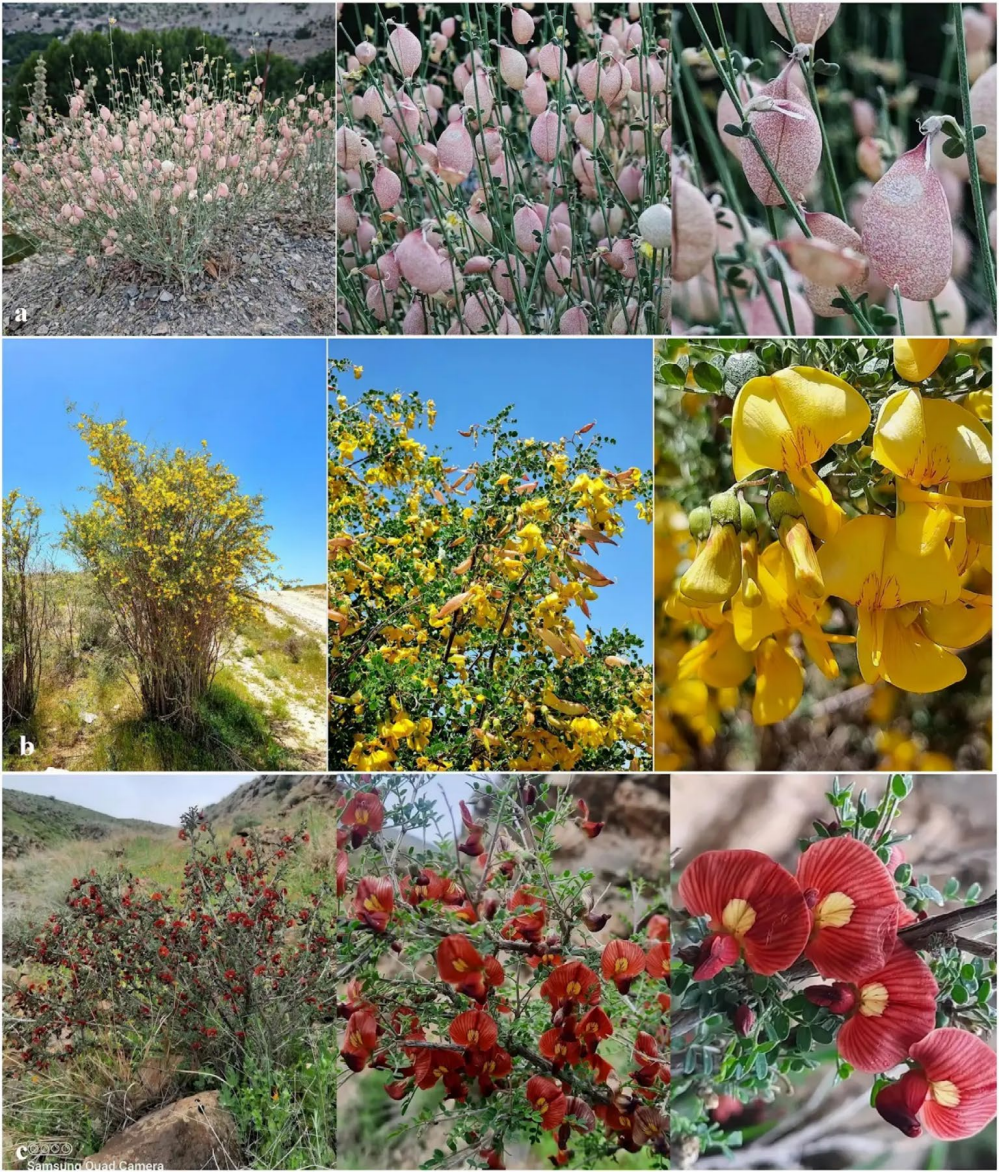
Photographs of *C. triphylla* (a), *C. persica* (b), and *C. porphyrogamma* (c) in natural habitats.

Among the woody taxa distributed in Iran, the genus *Colutea* (Fabaceae), commonly known as bladder senna, is composed of nearly nine species of shrubs and small trees in Iran, five of which are endemic to the country. Most of the species have been considered valuable shrubs, distributed in arid highlands, steppe, and semi‐steppe regions of the Iranian plateau (Boissier 1872; K. Browicz 1984; Kazempour Osaloo et al. 2006). Herein, we planned to assess the impact of climate change on the potential distribution of three *Colutea* species including *Colutea persica* Boiss., *Colutea porphyrogamma* Rech. f., and *Colutea triphylla* Bunge ex Boiss, which are endemic to Iran (Figure 1). These three species have been considered ornamental plants in their habitats due to possessing large and beautiful flowers and inflated legumes. *Colutea persica* has been known for its anti‐cancer effects against cancer cell lines, along with its application in traditional medicine as an anti‐inflammatory agent in gastrointestinal problems, purgative, emetic, and wound healing (Karimian et al. 2024; Hosseini et al. 2021; Mohamadi et al. 2015; Askari and Aghaabbasi 2022). The abovementioned importance of these species and the climate change pressures on their habitats motivated us to investigate the current and future spatial distribution of these taxa. For these *Colutea* species or similar taxa distributed in arid or semi‐arid regions, predictions of species occurrences derived from environmental suitability models can be used for practical conservation actions such as identifying and protecting critical habitats, reserve selection, determining potential recipient sites for reintroductions and translocation of endangered species to artificial fenced reserves, thereby contributing to preserving the genetic diversity of the species and guaranteeing their survival (Guisan et al. 2013; Riley et al. 2021).

In this context, species distribution models [SDM] have been used extensively in understanding the influence of climate change on the potential distribution of species and evaluating habitat suitability, not only for current conditions but also for future scenarios (Kamer Aksoy 2022; Hama and Khwarahm 2023). Among SDMs, the maximum entropy model (MaxEnt) has been recognized as one of the best performing methods compared to others when dealing with presence‐only data and has been extensively used to assess ecological requirements, environmental responses, and habitat suitability of species. MaxEnt is esteemed as a powerful algorithm for predicting species distributions, particularly in scenarios with few occurrence data such as endemic species, typically representing restricted distribution patterns (Elith et al. 2006; Elith et al. 2011; Phillips et al. 2006). This model illustrates the relationships between species distributions and environmental conditions across space and time, considering it among the most powerful tools for identifying appropriate habitats and predicting potential distributions of species (Ahmadi et al. 2023; Damaneh et al. 2022; Khajoei Nasab et al. 2022; Hosseini et al. 2024; Zeraatkar and KhajoeiNasab 2024).

We utilized the Maxent model in this research to (1) Model the current suitable distribution range of three *Colutea* species endemic to Iran, (2) Identify the most significant climatic and ecological variables influencing the distribution pattern of the studied species, and (3) Forecast the possible changes of habitat distribution for each species under proposed future climate change scenarios. In total, addressing these objectives will provide valuable information for decision‐makers concerning the future conservation management in the face of global climate change.

## 2 Materials and Methods

### 2.1 Study Area

The research area is restricted to the geographic boundaries of Iran, covering a total surface area of 1.65 million square kilometers, situated between 44°–64° E latitude and 25°–40° N longitude. Iran, as the second largest country in the Middle East, is located in the dry belt of Asia, and a variety of mountainous formations, such as Zagros, Alborz, Kopet Dagh, and Makran ranges, surround the central parts of Iran and prevent the penetration of humidity into the country (Zohary 1973; Noroozi et al. 2008). Therefore, diverse precipitation patterns appear in Iran, such as 800–2000 mm of annual rainfall in the northern slopes of the Alborz ranges, and the Caspian lowland receives 800–2000 mm of annual rainfall, in contrast to the Dasht‐e Kavir and Dasht‐e Lut deserts, located in arid regions, receiving less than 150 mm of annual precipitation. Meanwhile, the highland areas benefit from consistent rainfall, ranging between 250 and 800 mm (Jamshidi and Samani 2022). The elevation profile of Iran reveals remarkable extremes, ranging from −27 m a.s.l. in the Caspian basins to 5671 m a.s.l. at the peak of Mt. Damavand (the highest peak in Alborz). Iran also covers a varied range of soil types resulting from geological events, topography, climate, vegetation, and parent rock (Dewan and Famouri 1964).

### 2.2 Occurrence Data and Environmental Variables

The presence distribution data for three *Colutea* species in the study area were provided by a perfect assessment of Flora of Iran (Pooyan et al. 2023), Flora Iranica (K. Browicz 1984), and some other literature (K. Browicz 1963; Pooyan et al. 2014; Mirzaei et al. 2015), as well as historical records available in the herbaria of HSBU and D (the acronyms used in the study of Thiers 2023), and several floristic fieldworks in diverse regions of Iran during 2012–2022.

**FIGURE 2:**
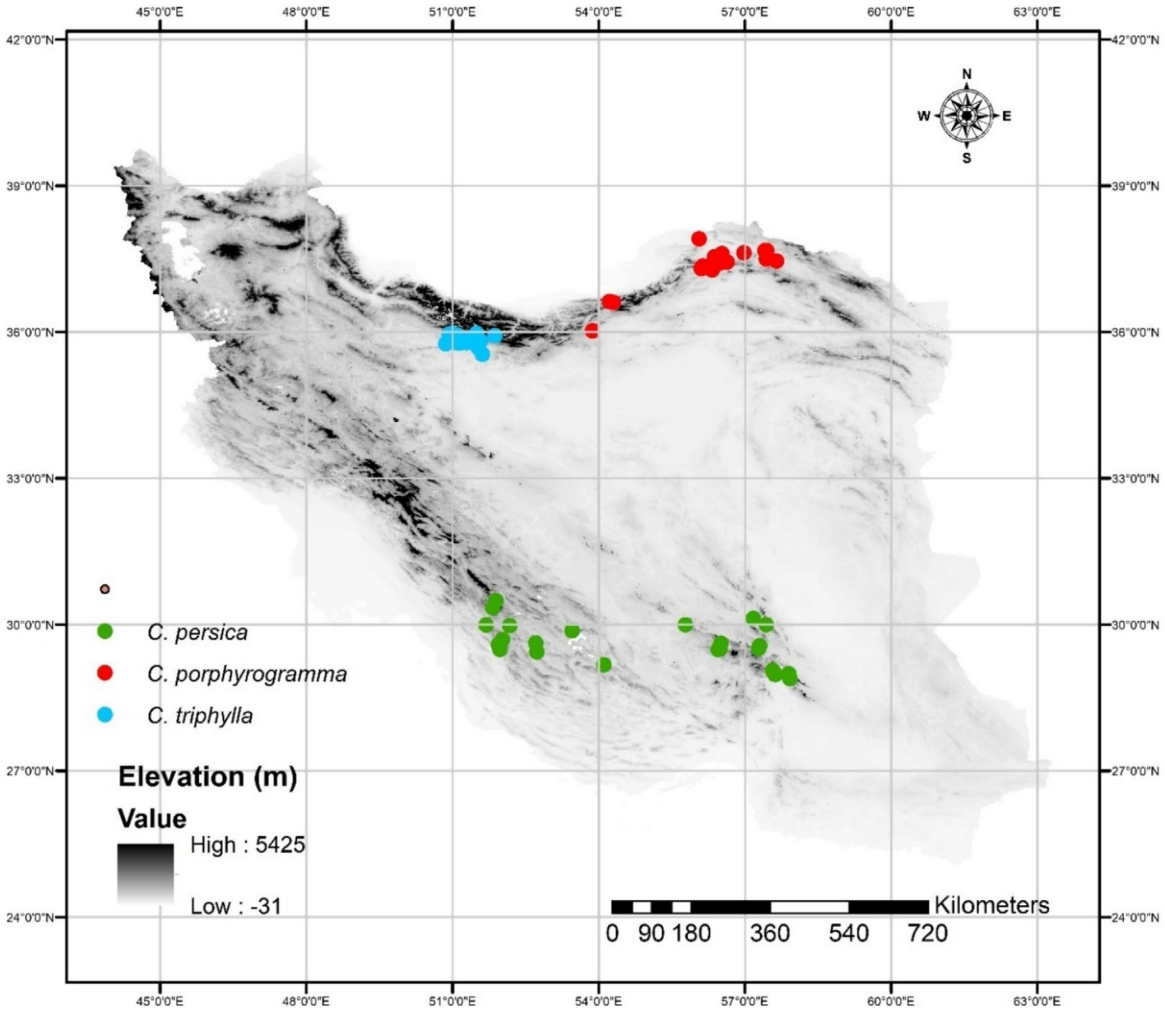
Occurrence data of *C. persica* , *C. porphyrogamma*, and *C. triphylla* in Iran.

As the modeling procedure used in this study (Maximum Entropy model) holds the potential to predict the species distributions with high accuracy even when lacking information about species absence (Phillips et al. 2006) and given that reliable absence data for these three *Colutea* species were not available, this study relied solely on presence data. In order to mitigate the spatial thinning among the occurrence records, any points with a separation distance of at least 1 km from one another was removed initially and before modeling. The distance 1 km, chosen for spatial thinning, is based on our field observations of the microhabitat range of each species and the environment of sampling. For removing duplicate points, the occurrence data was filtered by randomly selecting a presence point within a single grid cell (i.e., 1 × 1 km) using ‘sdm’ package in R environment (Ver. 4.2.2; Team 2013). After removal of such records, 26 occurrence data were selected for *C. persica* ; 21 occurrences for *C. porphyrogramma*, and 27 occurrences for *C. triphylla* (Data S1, Figure 2).

Afterwards, collinearity among environmental variables was tested by Pearson's correlation coefficient (*r*). If two variables were highly correlated (*r* > 0.70), one of them was excluded in order to avoid co‐linearity (Elith et al. 2011). Finally, guided by our observations on the ecological needs of each species during our field studies, available literature (K. Browicz 1963; K. Browicz 1984; Pooyan et al. 2014), and correlation test, a total of 11 variables were selected for *C. persica* , 12 variables for *C. porphyrogamma*, and 9 variables for *C. triphylla* . Current and future climate data, including 19 bioclimatic variables, was obtained from the WorldClim (https://www.worldclim.org/; Hijmans et al. 2017) and the raster layers of topographic variables and solar radiation were downloaded from https://www.Worldgrids.org. The Global Soil Grids (Soil Grids TM) database was used to prepare edaphic variables (https://www.isric.Org). For evaluating the future potential distribution of the target species, climate variables projected for the years 2050 (averaged from 2041 to 2060) and 2070 (averaged from 2061 to 2080) were taken into account. Furthermore, the CCSM4 climate model (Gent et al. 2011) was employed under both pessimistic (RCP 8.5) and semi‐optimistic (RCP 4.5) greenhouse gas emission scenarios. Among the various general circulation models (GCMs), CCSM4 stands out as one of the most effective tools for predicting the effects of impending climatic changes on the distribution of plants and animals in the Middle East (IPCC 2014). Initially, the layers were downloaded in GeoTIFF format from relevant global sources. We then imported these layers into ArcGIS software (Version 10.2) and used the Clip tool to adjust them to the scale of the study area. Following this, each variable was converted to ASCII format using the conversion tools, maintaining the geographic coordinate system GCS_WGS_1984. The resolution of the ecological variables employed was set at 30 arc sec, which is approximately equivalent to 1 by 1 km. Additional information can be found in Data S2.

### 2.3 Modeling Procedure

In this study, the maximum entropy algorithm (MaxEnt) was utilized to model the habitat suitability of the target species for both present and future scenarios. The implementation of the MaxEnt model, as outlined by Phillips et al. (2006), was carried out using MaxEnt v3.4.4 k (Phillips et al. 2006). This algorithm has been recognized as a high‐performance approach for predicting species distributions, particularly when working with limited sample sizes (Elith et al. 2006; Elith et al. 2011; Pearson et al. 2007). We employed 25% of species occurrence records for model testing and 75% for model calibration. The parameters for MaxEnt were set using the cross‐validation replicated run type, consisting of 10 replicates, a maximum of 1000 iterations, a convergence threshold of 0.0001, and 10,000 background points. The MaxEnt settings were based on the work of Kafash et al. (2022). They applied these settings to their model following the guidelines established by Merow et al. (2013). All settings were kept per the default MaxEnt configuration, except for the maximum iterations and the convergence threshold value. The convergence threshold was adjusted from the default value of 0.00001 to 0.0001, considered a reasonable value that can yield results close to the “real” response without excessive iterations. Additionally, the maximum iteration value was increased from 500 to 1000 based on the recommendation from Abrha et al. (2018). They suggested that increasing the maximum iterations allows the model sufficient time to converge, thereby minimizing the risks of under‐ and over‐prediction. We also considered permutation importance (PI) to identify the key environmental variables for MaxEnt models (Abdelaal et al. 2019). In interpreting machine learning models like Maxent, permutation importance is preferred over percent contribution due to its reliability and model‐agnostic nature. Permutation importance relies solely on the final Maxent model, whereas percent contribution assesses variable contribution by permuting values across training points, leading to potential variability based on the model structure. Consequently, many recent studies on species distribution modeling with Maxent utilize the importance of permutation to identify key variables (Abdelaal et al. 2024; Zeraatkar and KhajoeiNasab 2024). In order to evaluate how accurate our model results were, we applied a measurement called the area under the curve (AUC) associated with the receiver operating characteristic (ROC) curve. The AUC score ranges from 0 to 1.0, with a score of 0.5 indicating random prediction performance and a score of 1.0 indicating perfect performance. Scores below 0.50 indicate that the model is performing worse than random. To calculate the percentages of gain, loss, suitability, and unsuitability, we use the Intersect tool in ArcMap 10.2 and raster files generated by the WGS 1984 projection.

Finally, Suitability maps for each species under present and future climate scenarios were generated using ArcGIS software (Ver. 10.2).

## 3 Results

### 3.1 Evaluating the Model Performance and the Key Environmental Factors

**FIGURE 3:**
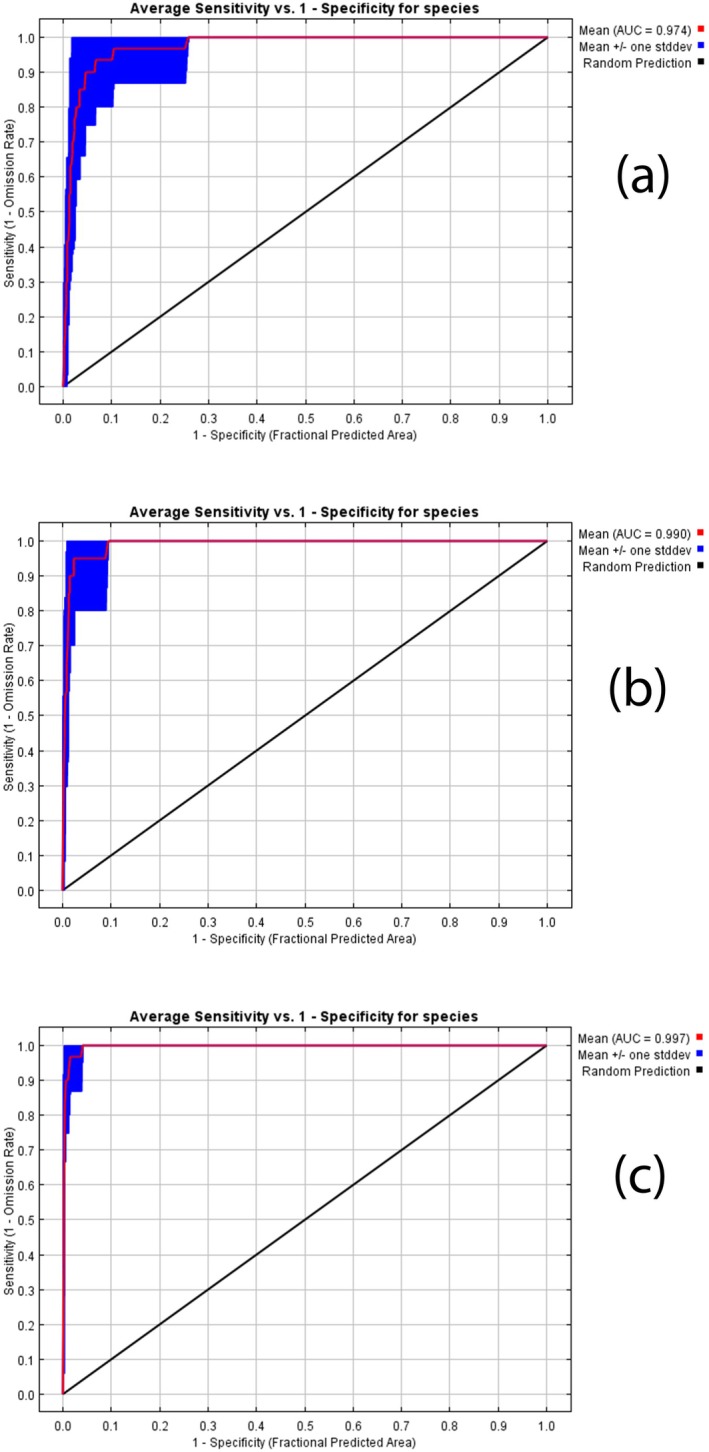
Graphical results of evaluating the model performance based on AUC values for *C. persica* (a), *C. porphyrogamma* (b), and *C. triphylla* (c).

According to the AUC values in our study, the modeling performance for three *Colutea* species was 0.98 for *C. persica* , 0.99 for *C. porphyrogamma*, and 0.99 for *C. triphylla* . Thus, our models represented an excellent level of prediction performance with AUC values in the studied species (Figure 3a–c).

**FIGURE 4:**
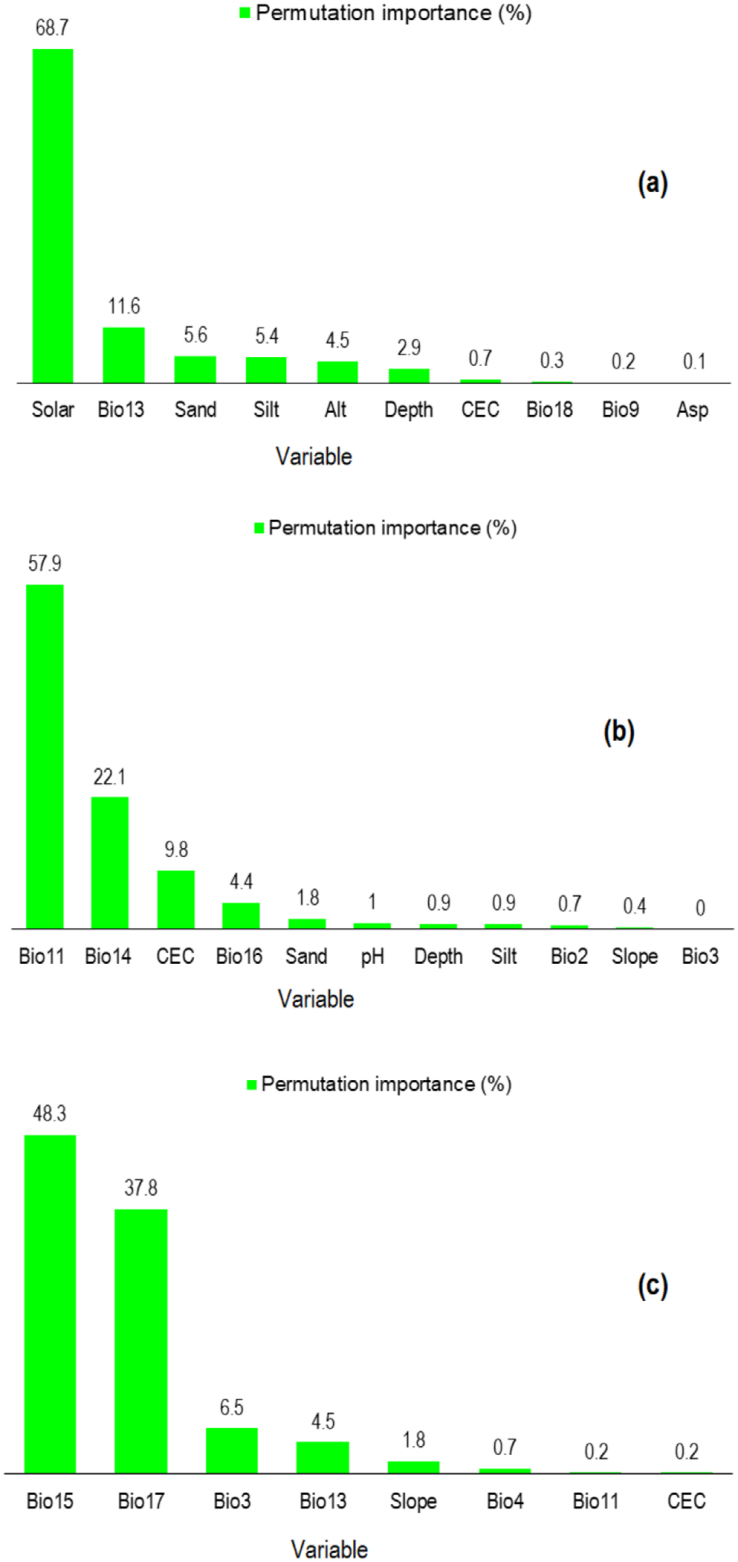
Percentage of permutation importance for environmental factors used in SDMs of *C. persica* (a), *C. porphyrogamma* (b), and *C. triphylla* (c) in Iran.

By analyzing the permutation importance of individual variables used in the modeling process, we found that the environmental factors contributing the most to the model for each species were different. The variables of solar radiation, Bio13 (Precipitation of Wettest Month), sand, and silt content had a significant impact on the potential distribution of *C. persica* (Figure 4a). The distribution of *C. porphyrogamma* was significantly impacted by Bio11 (Mean Temperature of Coldest Quarter), Bio14 (Precipitation of Driest Month) and CEC (Cation Exchange Capacity) (Figure 4b), while *C. triphylla* was most affected by Bio15 (Precipitation Seasonality), Bio17 (Precipitation of Driest Quarter) and Bio3 (Isothermality) (Figure 4c).

### 3.2 Potential Distribution of *Colutea* Species Under Current Climate Condition in Iran

**FIGURE 5:**
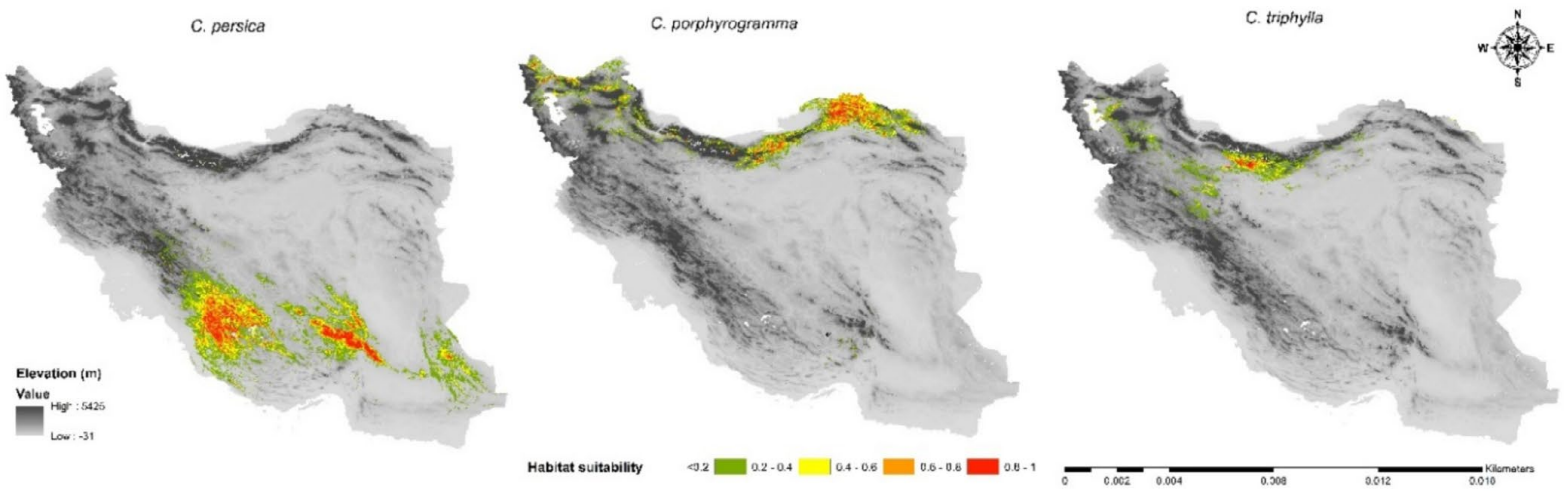
Map of potential current habitat suitability of the *Colutea* spp. in Iran.

The species distribution models under the current climate conditions represented that the highly suitable habitats for *C. persica* were predicted in the southern parts of Iran, specifically in Fars and Kerman provinces, and limited areas of Sistan and Baluchistan province (Figure 5). In the case of *C. porphyrogamma*, the potential suitable habitats were predicted in the Kopet Dagh–Khorassan region and Alborzian ecosystems as well as fragmented and dispersed areas throughout the Azerbaijan Plateau (Figure 5). Moreover, the model predicted that Alborz Mountain ranges are the best harbors of *C. triphylla* under current climate conditions (Figure 5). According to the MaxEnt model, *C. persica* had the largest suitable habitat area, covering 287,530 Km^2^ (17.44%), while *C. triphylla* had the smallest suitable habitat area, covering only 70,093 km^2^ (4.25%).

### 3.3 Climate Change Impacts on the Future Potential Distribution Pattern

In this study, the MaxEnt model‐based prediction for the future potential distribution of *C. persica* , *C. porphyrogamma*, and *C. triphylla* revealed that each species goes through a gradual transition in time zones and habitats (Figures 6, 7, 8, 9, 10, 11, 12, 13, 14).

**FIGURE 6:**
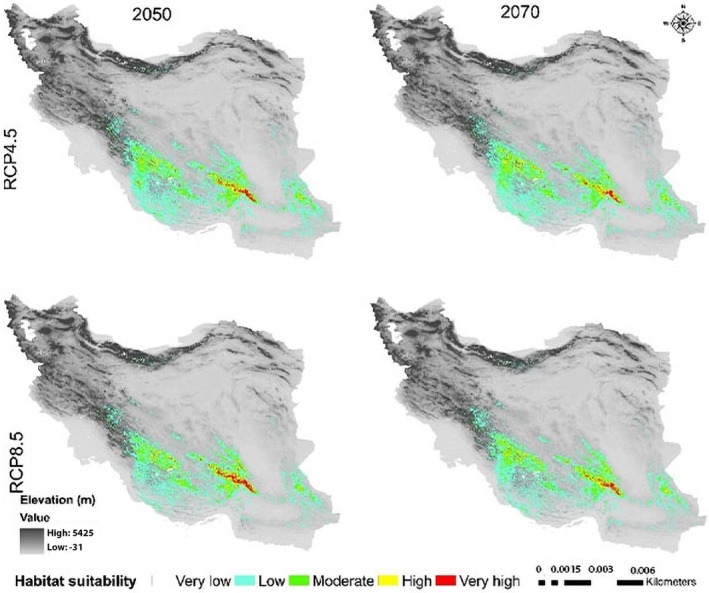
Potential range of *Crocus persica* in the future.

**FIGURE 7:**
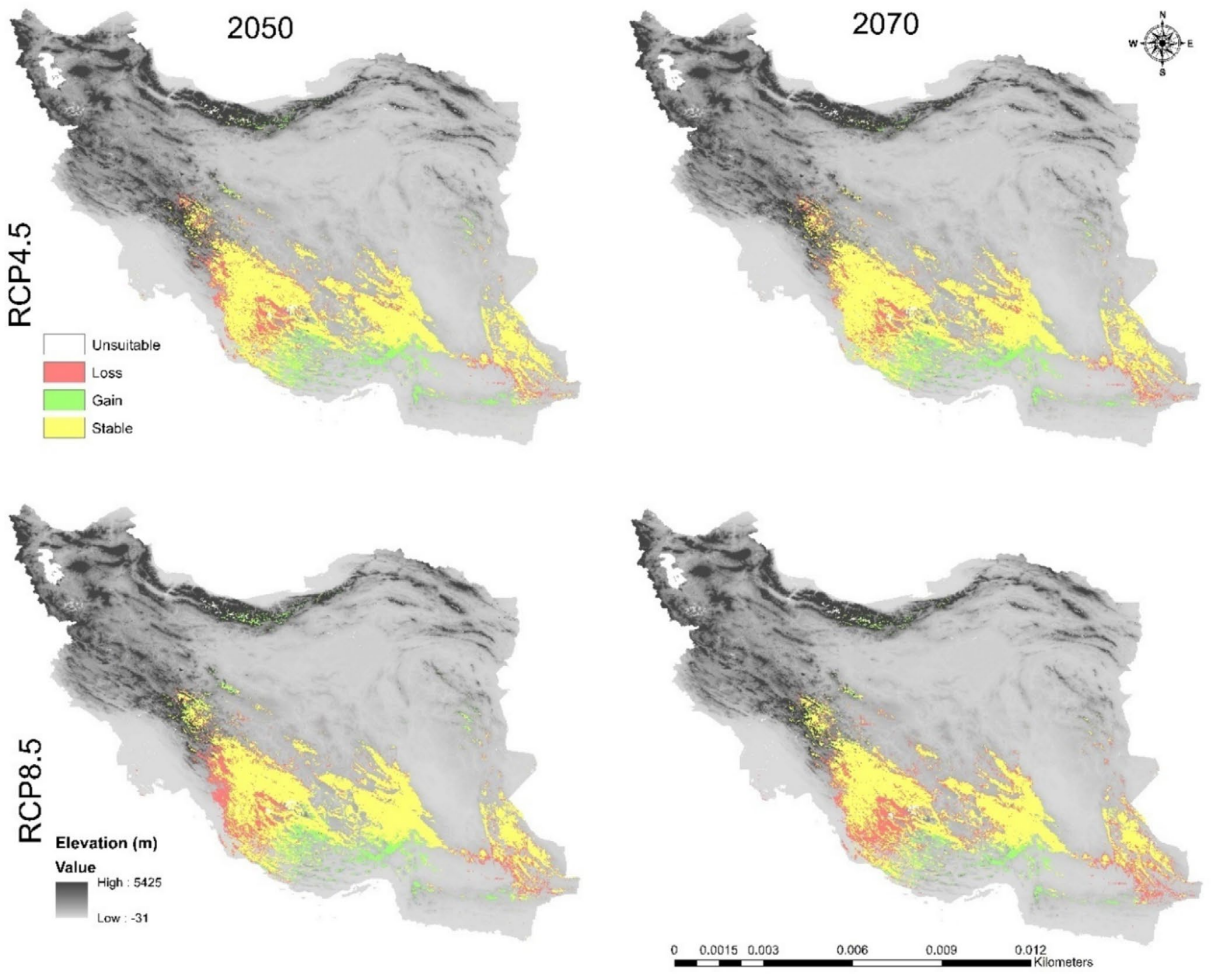
Range change between the current model of *C. persica* and the future models.

**FIGURE 8:**
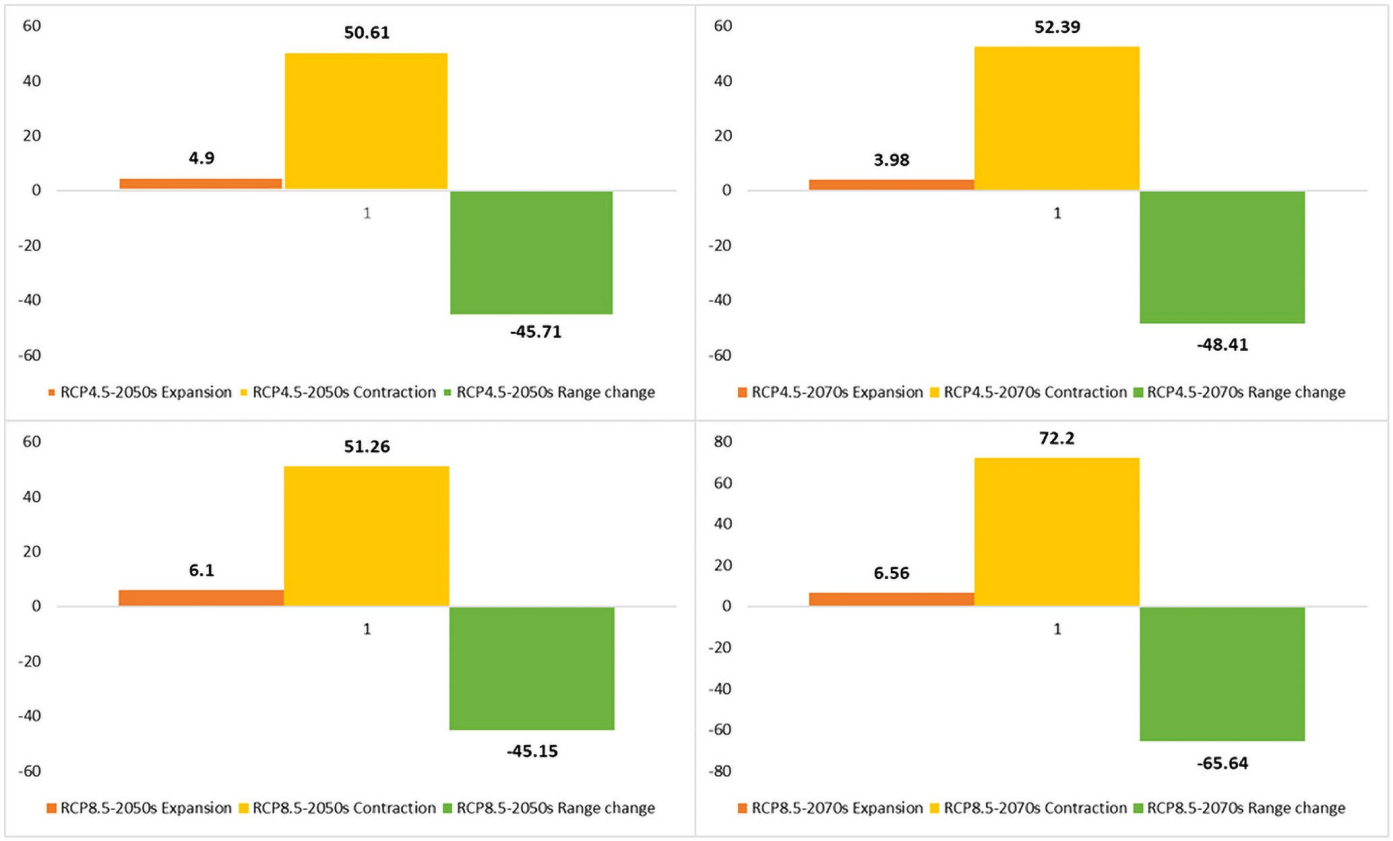
Percentage of the contraction, expansion, and range change of *C. persica* under pessimistic (RCP 8.5) and semi‐optimistic (RCP 4.5) greenhouse gas emission scenarios of the 2050s and 2070s.

**FIGURE 9:**
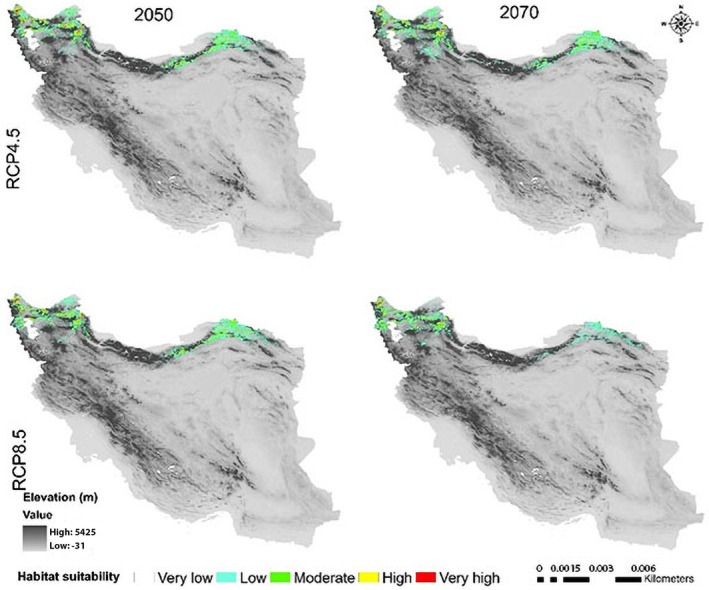
Potential range of *C. porphyrogamma* in the future.

**FIGURE 10:**
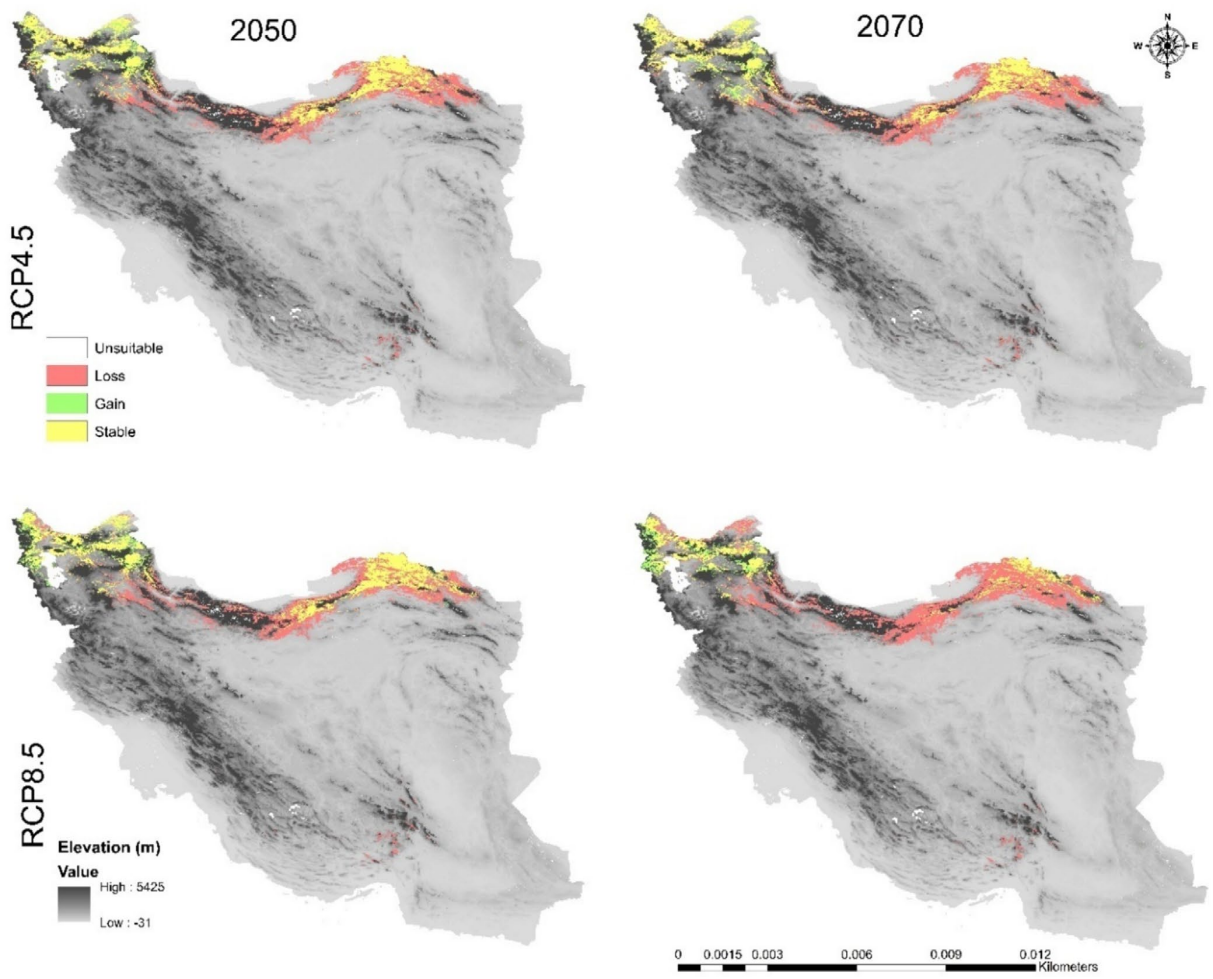
Range change between the current model of *C. porphyrogamma* and the future models.

**FIGURE 11:**
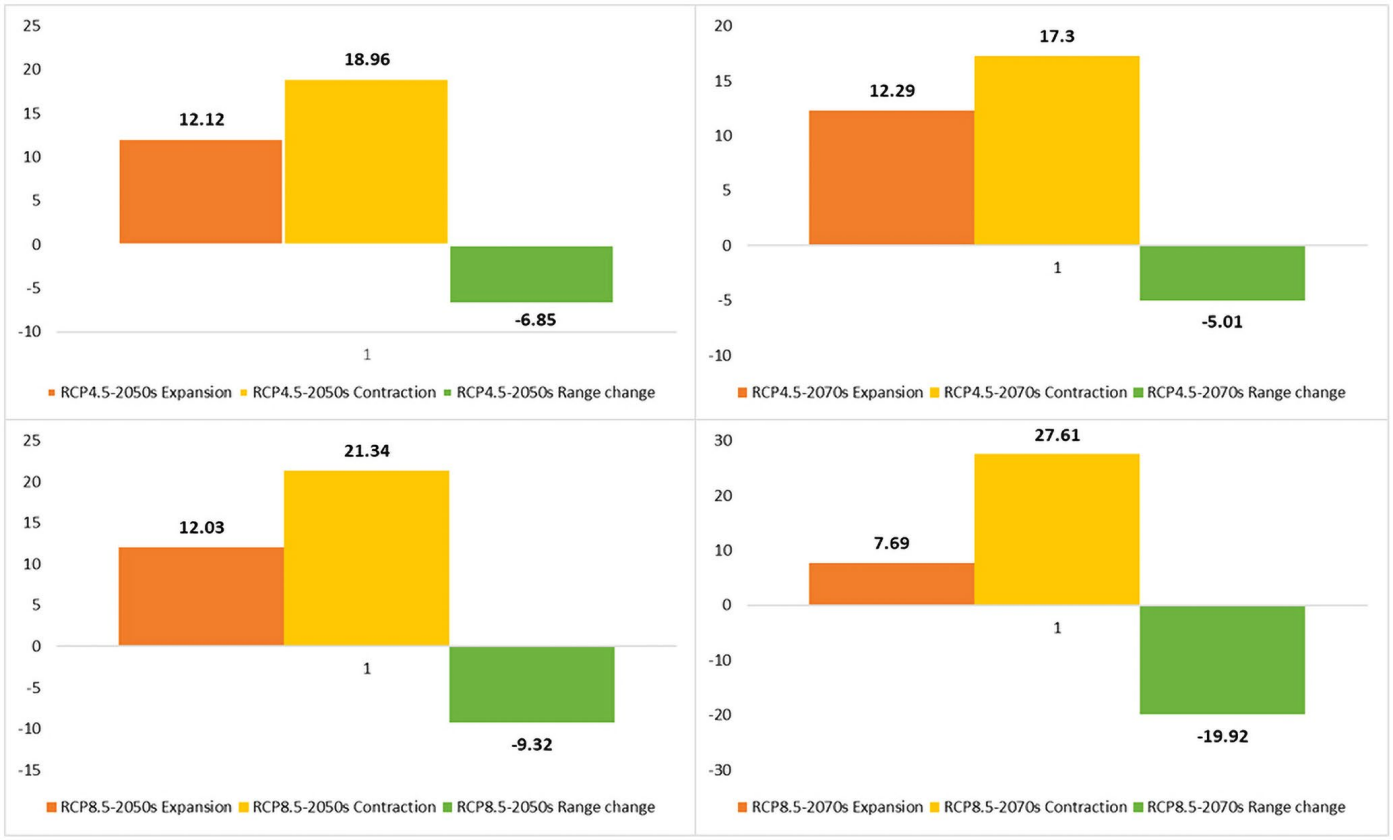
Percentage of the contraction, expansion, and range change of *C. porphyrogamma* under pessimistic (RCP 8.5) and semi‐optimistic (RCP 4.5) greenhouse gas emission scenarios of the 2050s and 2070s.

**FIGURE 12:**
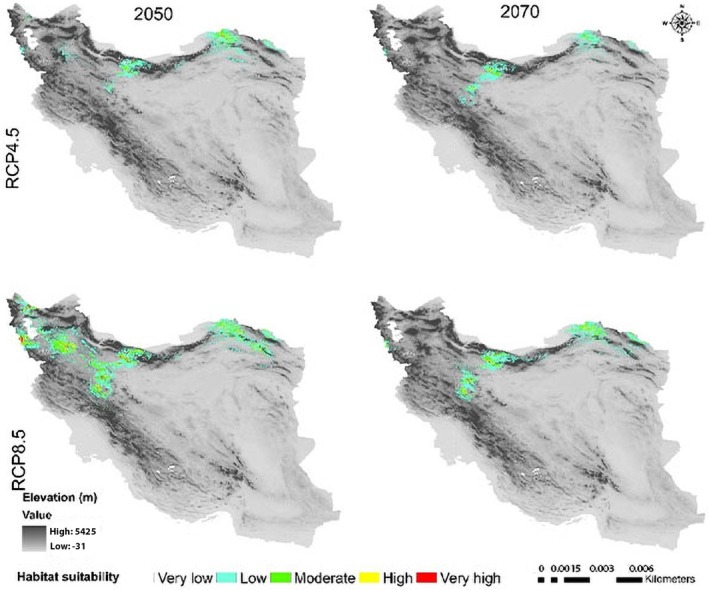
Potential range of *C. triphylla* in the future.

**FIGURE 13:**
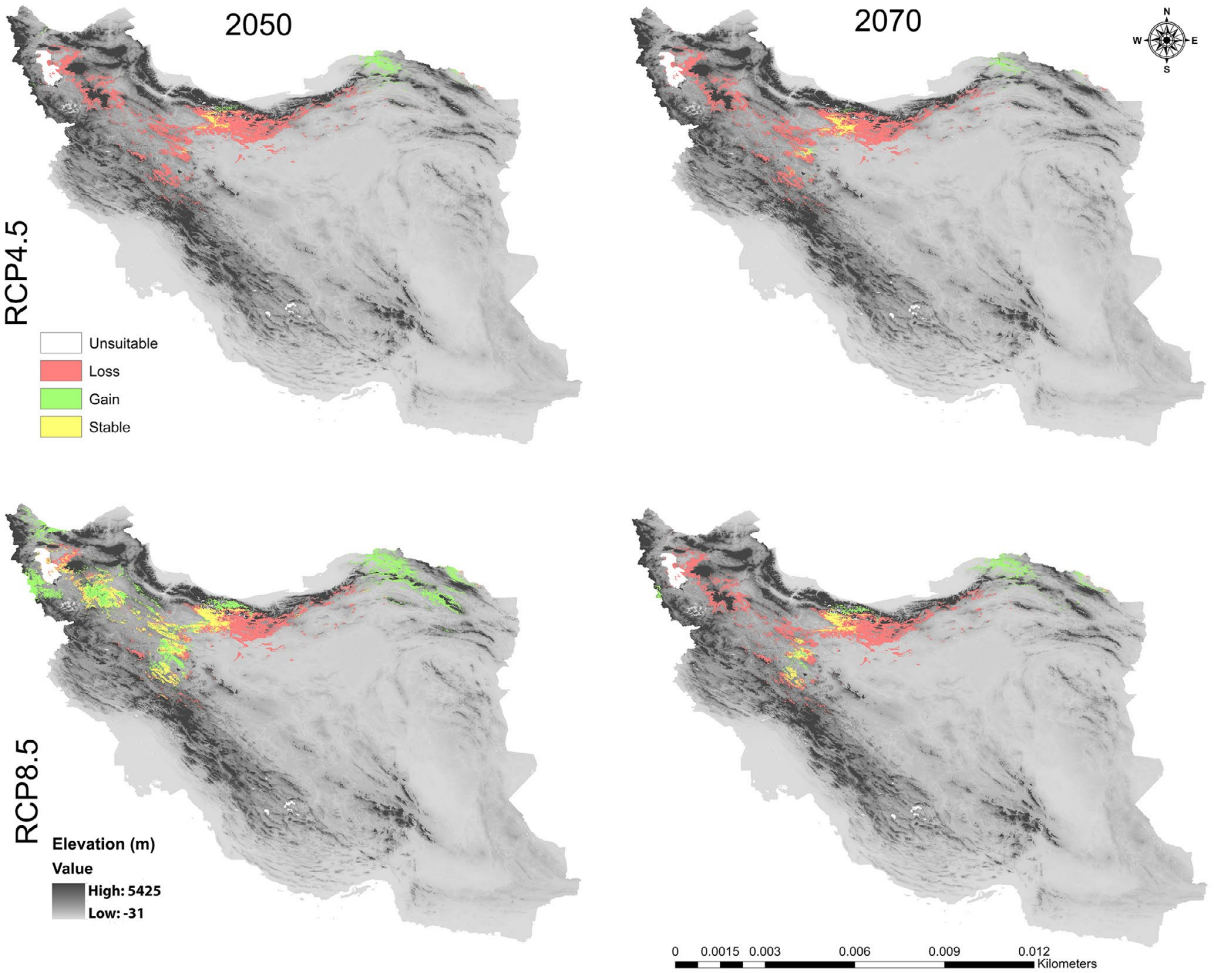
Range change between the current model of *C. triphylla* and the future models.

**FIGURE 14:**
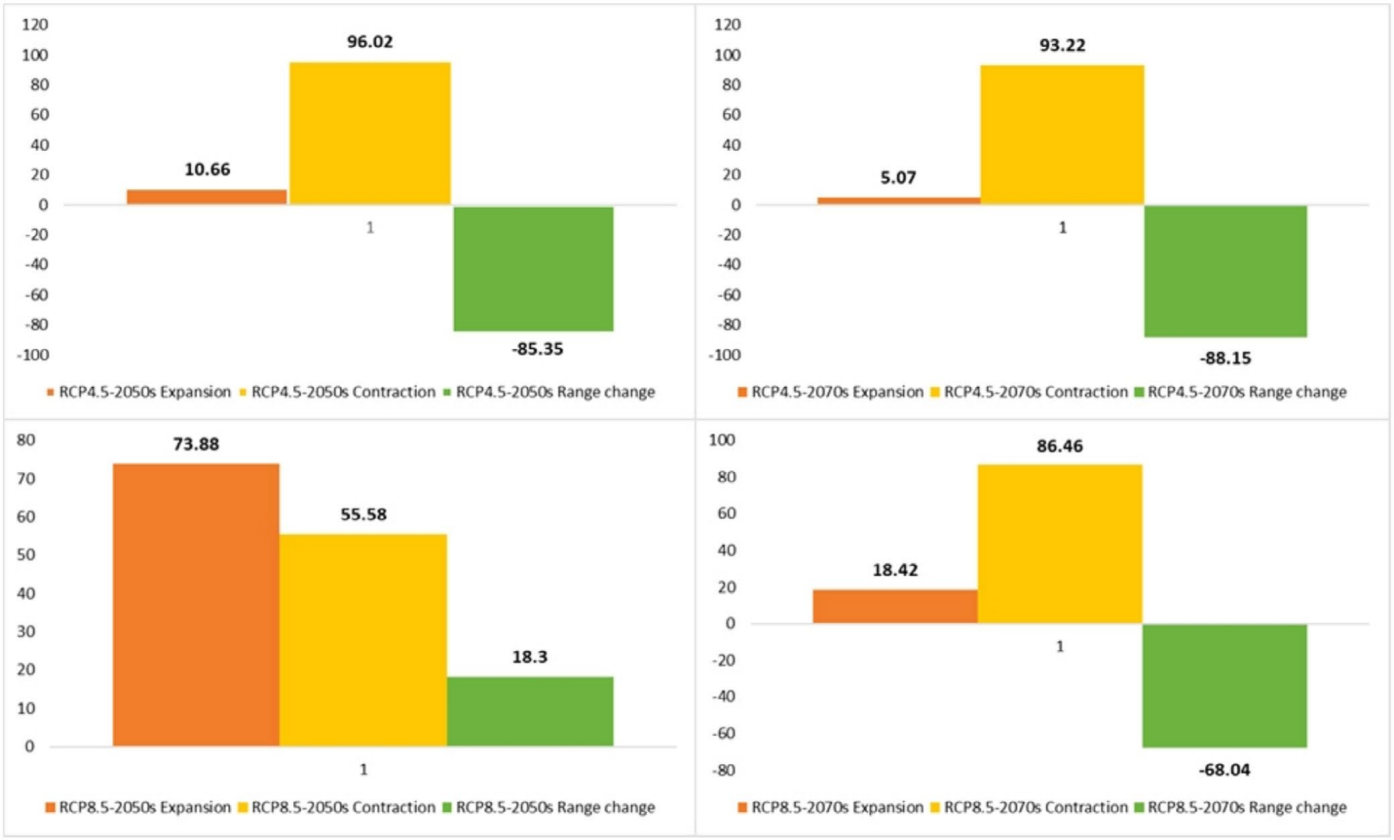
Percentage of the contraction, expansion, and range change of *C. triphylla* under pessimistic (RCP 8.5) and semi‐optimistic (RCP 4.5) greenhouse gas emission scenarios of the 2050s and 2070s.

The suitable habitat conditions predicted for *C. persica* and *C. porphyragamma* will decrease under the RCP 4.5 and RCP8.5 scenarios in the 2050s and 2070s (Figures 8, 11). Notably, the high suitability habitats for *C. persica* will be reduced in the highlands of Fars, Kerman, and Sistan and Baluchistan provinces (Figures 6, 7). For *C. Porphyrogamma*, the potential distribution range will decline in the Kopet‐Dagh Khorassan region and Alborzian ecosystems and will be restricted to high altitudes of the Azerbaijan Plateau (Figures 9, 10). Potentially suitable habitats for *C. triphylla* will decrease by 88.35% and 88.15% in the 2050s and 2070s under RCP 4.5, respectively, and will increase by 18.3% in the 2050s and decline by 68.04% in the 2070s under RCP 8.5 (Figure 14). The model predictions indicate that *C. triphylla* will be more vulnerable to future climate change than other studied species and will lose its desirable habitats in Alborzian ecosystems (Figures 12, 13); however, the total potential habitat for this species is projected to rise by 18.3% in the context of the 2050 RCP 8.5 scenario (Figure 14).

## 4 Discussion

Rising global temperatures are significantly impacting plant life cycles and geographic distributions, with a detrimental effect, particularly on woody plant species (Song et al. 2019; Abdelaal et al. 2019; Malanson and Alftine 2023). Our study emphasizes the susceptibility of *Colutea* species to the impacts of climate change. The findings indicate that the three *Colutea* species are expected to experience substantial decreases in their geographic ranges as a result of anticipated climate change projections. In this study, MaxEnt models based on future climate scenarios projected the highest risk of habitat loss for *Colutea triphylla*, followed by *C. porphyrogramma*, and then *C. persica* (Figures 6, 7, 8, 9, 10, 11, 12, 13, 14). These anticipated range contractions align with predictions for other temperate steppe montane shrublands across different regions globally (Giliba and Yengoh 2020; Adhikari et al. 2012; Adhikari and White 2016; Inague et al. 2021; Zhang et al. 2014).

Among the environmental variables, precipitation, soil conditions (sand, silt and CEC), and temperature were the most significant contributors to the distribution model for the *Colutea* species. These factors played a key role in controlling their distribution compared to other variables. Temperature and precipitation variables (Bio3, Bio11, Bio14, Bio15, Bio17) can be significant factors influencing the distribution of *C. porphyrogramma* and *C. triphylla* (Figure 4a,c). As far as these species predominantly grow in regions with cold and moderately moist conditions, their suitable habitats are mostly found in such areas (Memariani et al. 2016; Akhani 1998b; Akhani 1998b). On the other hand, MaxEnt outputs under current conditions indicated that *C. persica* distribution range was more influenced by solar and precipitation variables (Bio13, Figure 4a).

### 4.1 Precipitation Dominates the Current Distribution of Three *Colutea* Species

The *Colutea* species have predominantly expanded into relatively semi‐arid areas of the Irano‐Turanian region. Precipitation is regarded as the main factor affecting the growth and distribution of woody species in dryland ecosystems, particularly in areas with irregular rainfall patterns (Maestre et al. 2021). In these environments, where water availability is unpredictable and often scarce, the timing, amount, and frequency of precipitation events play a crucial role in determining the survival and growth of woody plants. In the rainy months, when heavy precipitation occurs, part of the rainwater infiltrates the soil from the surface and is directed to deeper layers of the earth, where it can be utilized by vegetation during the dry season. This process is particularly common in areas with permeable soils or where the soil has the capacity to absorb water (Zhang et al. 2016). Long‐term studies in arid regions show deep soil moisture is crucial for woody vegetation stability. Woody plants rely on deeper soil moisture, while herbaceous plants respond more to precipitation and water stress, with higher turnover. Precipitation variables such as the Precipitation of Wettest Month (Bio13) provide critical water resources during key growth periods, especially in climates where water is a limiting factor. Increased water availability during this period can enhance photosynthesis, nutrient uptake, and overall biomass production (Stringer et al. 2021; Wang et al. 2024). High precipitation during the wettest month often aligns with favorable conditions for seed germination and seedling establishment, particularly for species adapted to specific hydrological cycles. *Colutea porphyrogramma* and *C. persica* predominantly occur in high‐humidity microhabitats, such as moist valleys within their semi‐arid distribution range. These conditions provide sufficient moisture to support seed germination and seedling establishment, which are critical for their survival and regeneration (Noroozi et al. 2020; Memariani et al. 2016; Khodagholi 2017). It can define the boundaries of habitats, as many woody species are adapted to specific moisture regimes. Excess precipitation may lead to waterlogging, while its absence can limit survival. In ecosystems with variable water availability, woody species capable of utilizing the increased water during the wettest month may outcompete other vegetation types, shaping plant community structures. For woody species in dry regions, the wettest month's precipitation (Bio13) is critical to offsetting prolonged drought stress, replenishing soil moisture, and ensuring survival through dry periods. The Precipitation of the Driest Month (Bio14) can significantly affect woody species in various ways, particularly in ecosystems with seasonal or prolonged dry periods. Limited water availability during the driest month places woody species under significant water stress, affecting physiological processes like photosynthesis and transpiration. Shrublands with mechanisms to access deeper soil moisture are less affected than those relying on surface moisture in arid regions. The ability of woody species in almond and almond–pistachio communities to persist in areas with minimal precipitation during late spring and summer, when rainfall is absent, determines their geographic range and ecological niche (Khalili 2008; Pourmirzaee 2022).

*Colutea persica* is the only representative of its genus in the highlands and water‐abundant areas of the Zagros region, extending its distribution to the central mountains of Iran. The remaining species of this genus in Iran are confined to the Alborz Mountain range. Based on our study, Precipitation of the Wettest Month (Bio13) was one of the most influential factors in the habitat suitability of this species. As a result, factors related to water availability may have played a key role in its ecological adaptation and significantly influenced its distribution, as it is sensitive to drought. It typically grows at the edges of oak forests or in pistachio–almond woodlands, which receive high levels of rainfall (Figure 5). Consequently, the favorable growth areas for this species in the current map correspond to the high‐rainfall Zagros districts within the Irano‐Turanian floristic region, where we observe the growth of tree and shrub species in oak and Pistachio forests and woodlands of Pistachio–almond and almond. Interestingly, some areas of Sistan and Baluchistan province, including Mount Taftan, have been evaluated as highly suitable habitats for *Colutea persica* (Figure 5); therefore, these areas could serve as potential sites for introducing the species in the future. Tree and shrub species are typically sensitive to water stress, and these findings are consistent with other studies on shrubs like almonds (Choat et al. 2018; Zeraatkar and KhajoeiNasab 2024). Additionally, light is one of the most crucial growth factors for the distribution of *Colutea persica*. Solar radiation is a key element of ecological processes and influences the distribution, productivity, and composition of diverse ecosystems via photosynthesis and the hydrologic cycle (Piedallu and Gégout 2007). Many shrub species require high levels of light, as these plants are usually found at the edges of forests or areas of the forest where tree density is lower and more light reaches the floor. In this genus, the species such as *Colutea cilicica* Boiss. & Balansa, *C. arborescens* L., *C. porphyrogramma*, *C. buhsei* (Boiss.) Shap., *C. gifana* Parsa, and *C. istria* Mill. are all shrubs. These species cannot compete with forest tree species and are therefore found at the edges of oak or pine forests (K. Browicz 1963; Özdemir and Çınar 2023). Consistently, previous studies have shown that the distribution of woody species is profoundly affected by solar radiation (Bayat et al. 2021; Piedallu and Gégout 2007; Zeng et al. 2014).

*Colutea porphyrogramma* is a rare and endemic shrub, mainly distributed in a transitional position of bioclimatic zones of Central Iranian deserts of Irano‐Turanian region, Karakum Desert, and the Hyrcanian province of Euro‐Siberian region (Figure 5). Since the variables of precipitation (Precipitation of Driest Month [Bio14] and Precipitation of Wettest Quarter [Bio16]) are important for the species distributed in Alborz Mountains, increased precipitation seems to be a relatively limiting factor at the northern border of the current distribution of *C. porphyrogamma*, as it is unable to penetrate the dense and highly humid Hyrcanian forests due to soil factors. Similarly, Bio14 had a substantial contribution to the projected distribution of woody species such as *Buxus hyrcana*, *Hedera pastuchovii*, and *Ruscus Hyrcanus* (Sękiewicz et al. 2024). *Colutea porphyrogamma* is predominantly distributed in semi‐mesic areas (300–450 mm) such as *Juniperus polycarpos* K. Koch woodlands, open successional scrub communities including *Acer monspessulanum* L., *Crataegus* spp. and *Paliurus spina‐christi* Mill. scrubs and also *Artemisia* and *Artemisia‐Stipa* steppes (Memariani et al. 2016; Jafari and Akhani 2008; Akhani 1998a; Akhani 1998b).

*Colutea triphylla* is a subshrub species with a limited distribution, primarily found on the southern slopes of the central Alborz mountain range in Iran (Figure 5). The highly restricted distribution of *Colutea triphylla* to a small area of the central Alborz Mountains indicates that this species is a narrow endemic. This conclusion is further supported by past field surveys, herbarium records, and published literature, as all of which confirmed the species is not found outside this area. Precipitation and temperature variables such as Bio15 (Precipitation Seasonality), Bio17 (Precipitation of Driest Quarter) and Bio3 (Isothermality) played a key role in determining the distribution of potential habitats of *C. triphylla* in its native range. Bio3 is the mean daily temperature range (monthly mean) to the annual temperature range with a positive value. It demonstrates that a stable temperature environment was beneficial for the growth of *C. triphylla* . Accordingly, it was reported that these environmental variables (Bio3, Bio15) play an important role in the geographic distribution of some woody species such as *Taxus baccata* L. in the Hyrcanian Forest of Iran (Ahmadi et al. 2020) and *Tapiscia sinensis* Oliv. in China (Xie et al. 2023). Model output and field surveys revealed that suitable natural habitats of the species concurred with the distribution of sub‐humid temperate climate in the southern slopes of the Alborz Mountains. Some areas of the northern Zagros Mountains were evaluated as highly suitable habitats for *Colutea triphylla*, offering potential for future introduction. In other words, the suitable habitats of *C. triphylla* are mainly concentrated in the central Alborz, with some presence in the northern parts of the Zagros region, where moisture levels are relatively high (Figure 12).

### 4.2 Distributions of Three *Colutea* Species Shift Across Higher Latitudes and Altitudes

Root et al. (2003) investigated the impact of global climate change on distribution ranges of various species and found that the distributional shifts of more than 80% of the species were consistent with the expected biological responses to changing climates. These shifts mainly entailed movements toward higher latitudes or elevations, prompted by adaptations to microclimate conditions that are crucial for the survival of populations.

The outcomes of our SDMs revealed that three *Colutea* species are forced to shift their ranges upwards to higher latitudes or altitudes (south or north) with cooler and more humid environments, as far as the climate gets warmer and lower elevations become drier. Based on our results, the three endemic *Colutea* species in Iran will shift toward other phytogeographic boundaries in search of suitable habitats due to predicted changes in climate. These areas offer cooler temperatures and higher rainfall, which will increase their chances of survival (Wiatrowska et al. 2020). It was also confirmed in previous studies that plant species sensitive to global warming may shift their range to cooler locations at higher altitudes or latitudes (Shamsabad et al. 2018; Paź‐Dyderska et al. 2021; Song et al. 2019; Zhang et al. 2020).

Low‐altitude areas, such as plains and deserts in central Iran and the Saharo‐Sindian regions, will become less suitable for the survival of *C. persica* , owing to low rainfall. Furthermore, the dense oak forests and alpine regions of the Zagros Mountains will hinder the growth of any subshrubs and make their upward shift impossible (Zeraatkar and KhajoeiNasab 2024). Concerning the RCP 4.5 and RCP 8.5 scenarios for *C. persica* , it was anticipated that this species will migrate southward to the low‐altitude mountains of southern Iran in the Saharo‐Sindian floristic region (Figure 7). Consistent with previous studies, there are various examples of different species distributed between the Irano‐Turanian and Saharo‐Sindian regions, such as *Haplophyllum canaliculatum* Boiss., *Scutellaria ariana* Hedge, *Matthiola longipetala* (Vent.) DC., *Hyoscyamus tenuicaulis* Schönb.‐Tem., and *Moluccella aucheri* (Boiss.) Scheen, etc. (Noroozi et al. 2020).

For *Colutea porphyrogramma*, many of its current habitats will be lost in the future because of rising temperatures and decreasing rainfall. Furthermore, this species will not be able to establish itself in regions adjacent to current distributional areas like the Dasht‐e Kavir in Iran or the Karakum Desert in Turkmenistan, since these areas are too dry and hot (Noroozi et al. 2020). Based on all future emission scenarios, *C. porphyrogramma* will shift toward the northwest, migrating from the mountains of Kopet Dagh‐Khorassan province to the mountains of Azerbaijan Plateau, where the colder and wetter climate will be more conducive to its survival (Figures 9, 10). It is also worth mentioning that northwest of Iran is one of the diversity centers for the genus *Colutea*, besides the South Caucasus and eastern Turkey, with several species of this genus spreading throughout these regions (K. Browicz 1963).

The results of both RCPs scenarios indicated that *C. triphylla* will lose almost all of its current suitable habitats and instead move toward the Azerbaijan Plateau and the Kopet Dagh–Khorassan floristic provinces, as well as the northwestern part of the Zagros Mountains, where more water is available (Figures 12, 13). This decline in habitat suitability of such narrow‐ranging species would result from their inability to adapt to changes in climate conditions such as rising global temperatures and decreasing precipitation (Jiang et al. 2022; Dubos et al. 2022).

### 4.3 Importance of Soil Variables in the Distribution of *Colutea* Species

In addition to climatic changes, other factors such as soil composition would also contribute (Hulshof and Spasojevic 2020). The distribution of many legume species is strongly influenced by soil conditions, including factors such as soil texture, nutrient content, pH, and moisture availability (Baptista et al. 2020). The species of *Colutea* are commonly found in soils with a high content of CEC (Nadaf et al. 2017). The association between legumes and soil rhizobia is an example of a highly specialized mutualistic interaction within the family Fabaceae. Most legumes form mutual associations with bacteria from the group of alpha‐proteobacteria and beta‐proteobacteria, which play an important role in agriculture, particularly in connection with biological nitrogen fixation (Liu et al. 2018). Commonly known as rhizobia, these bacteria enable legumes to access atmospheric nitrogen, which is typically unavailable to most plants. Through their interaction with legumes, Rhizobium bacteria indirectly contribute to increasing the cation exchange capacity (CEC) of soils. While the bacteria themselves do not directly enhance soil CEC, their symbiotic relationship promotes conditions that improve organic matter and soil health, ultimately leading to higher CEC over time (Traoré et al. 2012; Wang et al. 2023; Alon et al. 2024).

In line with other studies that have explored the distribution of woody species, our results demonstrate that the distribution of two shrubby species, *Colutea persica* and *C. porphyrogramma*, is also influenced by edaphic factors, including sand and silt content, as well as soil depth (Comole et al. 2021; Gxasheka et al. 2023). Soil texture and depth are important to plant growth of tree and shrub species as they influence water availability and soil fertility, i.e., the ability of the soil to deliver nutrients to plant roots (Rajakaruna and Boyd 2008). Coarse‐textured soils (such as sandy and silty soils), in particular, enhance the growth of woody plants and allow for increased water infiltration and nutrient leaching to deeper soil layers, which supports the growth of woody plants (Zeraatkar and KhajoeiNasab 2024). Moreover, coarse‐textured soils facilitate rapid water drainage from the surface layers, thereby hindering the upward movement and evaporation of water from the lower soil layers. This implies that coarse‐textured soils are more advantageous for deep‐rooted woody plants, as these plants can access and utilize soil moisture from greater depths. To outcompete herbaceous species, woody species extend their root systems deeper into the soil, taking advantage of moisture reserves stored in the deeper soils typically found on lower slopes (Hulshof and Spasojevic 2020). Consistently, several ecological modeling studies represented the major contribution of this soil type on shrub expansion; for instance, Browning et al. (2008) and Comole et al. (2021) showed the significant influence of sandy soil on the distribution of *Prosopis* L. shrubs in Australia and South Africa, respectively.

## 5 Conclusions

This study highlights the urgency of protecting three *Colutea* species endemic to Iran. These species are significant since they are unique to Iran and cannot be found anywhere else in the world. They play a crucial role in combating various types of erosion, such as soil erosion, as well as preventing floods, and ultimately contribute to climate stabilization. It was also revealed that the studied species are sensitive to climatic factors, particularly rainfall, which significantly limits their distribution. Therefore, it is likely that droughts caused by global warming will pose a serious threat to the survival of these valuable plant species. The results indicated that the species *C. triphylla* is the most endangered of the studied species, highlighting the need to develop and implement specific strategies for its protection. Additionally, the models predicted that these species will migrate to more northern regions in response to climate change. While this may help some populations of these species escape extinction, it could also disrupt ecosystems by invading new environments and competing with existing species. It appears that the results of species distribution modeling can be beneficial for the management and protection of plant species; therefore, we recommend continuous monitoring and observation of these *Colutea* species over time. Considering that drought, fire, indiscriminate harvesting, and the spread of pests are major threats to many forests in the country, the vegetation of Iran, especially the flora of woody species, is at risk of complete destruction in the coming decades. Hence, we propose to expand such type of research to other plant species, giving priority to endemic woody species which are at risk of extinction in Iran.

## Author Contributions

**Amin Zeraatkar:** conceptualization (equal), project administration (equal), resources (equal), writing – original draft (equal). **Elham Hatami:** data curation (supporting), project administration (supporting), writing – original draft (equal), writing – review and editing (equal). **Farzaneh Khajoei Nasab:** conceptualization (equal), data curation (lead), formal analysis (equal), methodology (lead), visualization (equal), writing – review and editing (equal). **Najmaldin Ezaldin Hassan:** data curation (supporting), resources (supporting), writing – review and editing (supporting).

## Conflicts of Interest

The authors declare no conflicts of interest.

## Supporting information


**Data S1.** Occurrence records of *Colutea persica*, *Colutea porphyrogamma*, *Colutea triphylla* in Iran.

**Data S2.** Environmental variables related to the distribution of three *Colutea* species in Iran.

## Data Availability

All the required data are uploaded as Data S1 under the file “*Colutea* occurrence data” and Data S2 under the file “Environmental variables”.
